# Advances in neural organoid systems and their application in neurotoxicity testing of environmental chemicals

**DOI:** 10.1186/s41021-021-00214-1

**Published:** 2021-09-22

**Authors:** Yuanyuan Zheng, Fangrong Zhang, Shengmin Xu, Lijun Wu

**Affiliations:** Information Materials and Intelligent Sensing Laboratory of Anhui Province, Institutes of Physical Science and Information Technology, Anhui University, Hefei, 230601 China

**Keywords:** CNS, hPSC, Neural organoids, Neurotoxicity testing, Environmental chemicals

## Abstract

Due to the complex structure and function of central nervous system (CNS), human CNS in vitro modeling is still a great challenge. Neurotoxicity testing of environmental chemicals mainly depends on the traditional animal models, which have various limitations such as species differences, expensive and time-consuming. Meanwhile, in vitro two-dimensional (2D) cultured cells or three-dimensional (3D) cultured neurospheres cannot fully simulate complex 3D structure of neural tissues. Recent advancements in neural organoid systems provides excellent models for the testing of environmental chemicals that affect the development of human CNS. Neural organoids derived from hPSCs not only can simulate the process of CNS development, including early stage neural tube formation, neuroepithelium differentiation and regional specification, but also its 3D structure, thus can be used to evaluate the effect of chemicals on differentiation and morphogenesis. Here, we provide a review of recent progress in the methods of culturing neural organoids and their applications in neurotoxicity testing of environmental chemicals. We conclude by highlighting challenge and future directions in neurotoxicity testing based on neural organoids.

## Background

If fetus exposed to industrial chemicals in the environment, the development of central nervous system (CNS) might be damaged, and thus neurodevelopmental disorders and birth defects will occur, such as attention deficit hyperactivity disorder (ADHD), mental retardation and neural tube defects. There is increasing evidence that exposure to endocrine-disrupting chemicals, particularly phthalates and bisphenol A, may be associated with ADHD [1, 2]. Even exposure to low environmental toxins such as lead and mercury can have subclinical effects on the brain, such as mental decline or behavioral changes [3, 4]. Neural tube defects (NTDs) are a kind of severe congenital CNS defects, including common subtypes such as anencephalia and spina bifida. They are the main causes of perinatal death and lifelong disability. Until now, it has been recognized that traditional pollutants such as heavy metals, pesticides, organic solvents and radiation are environmental risk factors for NTDs. For example, research found that Cd may be a risk factor for NTDs, and the risk effect may be enhanced in fetuses who carry the G allele of rs4880 in SOD2 and T allele of rs1801133 in MTHFR [5]. Previous study also indicated that the levels of polycyclic aromatic hydrocarbons and some organochlorine pesticides in placenta were associated with the risk of neural tube defects, and showed a significant dose-response relationship [6]. However, due to the ethical problems of in vivo research on human embryos and the complexity of CNS development, the neurotoxicity mechanisms of these environmental factors has not been fully understood [7]. In addition, with the development of industry, the types and quantity of chemicals in the environment increase rapidly. Currently, there are more than 40,000 chemicals registered in China, however, human CNS neurotoxicity testing for most of these chemicals are still lack of sufficient evidence. It reported that new environmental chemicals such as perfluorinated compounds and bisphenol compounds have been detected in pregnant women [8]. Tetrabromobisphenol S, a halogenated flame retardant widely used in daily necessities, has also been detected in breast milk and umbilical cord blood [9]. Nevertheless, neurodevelopmental toxicity and mechanisms of these new environmental chemicals are still unclear.

Traditionally, neurotoxicity testing has been based on animal models such as mice, rats, zebrafish and rabbits, which are regularly used to develop human exposure guidelines. By animal models, the researchers preliminarily explored the toxic effects of environmental pollutants on the development of CNS [10]. Ayelet et al. proved that carbamazepine residue in wastewater could affect the morphology, closure, cell proliferation and differentiation of neural tube, resulting in NTD through chicken embryo model [11]. Although animal model is a classic method to study the neurodevelopmental toxicity of environmental pollutants, this method has ethical issues and being expensive, time-consuming, and labor-intensive, making it impossible to conduct neurotoxicity testing on all commercially used chemicals. More importantly, at the structural, cellular and molecular levels, there are significant differences in the CNS between animals and humans. Thus, the toxic mechanism of chemicals to humans may be different from that of animals, which makes it difficult to extrapolate the evaluation results from animals to humans [12]. Therefore, it is necessary to develop rapid and low-cost in vitro methods for the detection of potential neurodevelopmental toxicity chemicals based on human cells derived from human donors [13].

Human pluripotent stem cells (hPSCs), including human embryonic stem cells (ESCs) derived from blastocysts and induced pluripotent stem cells (iPSCs) produced from somatic cells, have the capacity to proliferate extensively and differentiate into multilineages. The two-dimensional (2D) culture model from hPSCs has become an important way to study neurodevelopmental toxicity. However, human CNS tissue is a very complex 3D structure composed of cells and extracellular matrix, 2D cell culture or 3D cultured neurospheres cannot reflect the complete tissue structure. It is impossible to study the effect of chemicals on the structural changes of CNS tissue by 2D cell culture model, therefore, researchers turned to hPSCs derived 3D neural organoids to investigate the neurotoxicity effects of environmental chemicals. Human neural organoids are distinct from traditional neurospheres and 3D tissue engineered constructs consisting of cells seeded on a 3D scaffold. As defined by Lancaster and Knoblic, organoids are 3D cell complex formed by inducing differentiation of hPSCs or organ progenitor cells and self-organizes through cell sorting and spatially restricted lineage commitment in a manner similar to in vivo [14]. Initially, neural organoids models mainly refer to brain or specific-region brain organoids, but recently, rapid progress has been made in the modeling of spinal cord, neuromuscular junction and corticospinal tract. At present, researchers have extended the term neural organoid to spinal cord organoid [15]. Therefore, neural organoid can recapitulate the structure and function of CNS in vitro, emerged as a new experimental modality to study early signaling processes that are essential to brain and spinal cord development, function, and disease.

In this review, we analyze the various neural organoids generation protocols from hPSCs and how these models have been used in neurotoxicity testing research. Finally, we discuss challenges for the development of advanced organoid-based in vitro systems for neurotoxicity testing.

## Overview of early stage CNS development

The development of vertebrate CNS can be divided into three stages: neural induction, patterning formation and neurogenesis. The process of notochord inducing the formation of neural tube is neural induction. Firstly, the dorsal ectoderm is differentiated into neural plate, which is composed of neuroepithelial cells, under the action of neural inductive factors. Then, the edge of neural plate became thicker and uplifted to form nerve fold. Finally, the neural folds fuse in the dorsal midline to form a hollow neural tube, in which the neuroepithelial cells remain undifferentiated, and constantly expand the number of cells through rapid proliferation [16]. Environmental toxicants interfere with these processes will lead to neural tube defects.

After the formation of neural tube, it patterns along anterior posterior (AP) axis and dorsal ventral axis to guide the expression of development related genes in specific cell groups of neural axis. For anterior posterior axis patterning, the traditional view of neural development is that the anterior neural plate is first produced under the action of activation signal. Then, the mesoderm at the relatively late development stage provides “transforming signals”, for example retinoic acid (RA), inducing anterior neural plate to produce midbrain, hindbrain and spinal cord located in the posterior of neural axis. This model is called “activation transformation” model, which suggests that the posterior nervous system, such as the spinal cord, acquires the anterior fate of the neural axis prior to the posterior fate [17, 18]. However, in recent years, a new view has been put forward based on research of model animals, that is, there are two ways for early epiblast cells to obtain neural fate. The first is to produce anterior neural plate through ectoderm, and then develop into anterior neural tissue, including forebrain, midbrain, hindbrain and anterior spinal cord. The other is to produce neural mesodermal progenitors (NMPs) through mesoderm, and then develop into posterior spinal cord [19, 20]. NMPs are both positive for neural precursor cell marker Sox2 and mesoderm precursor marker Brachyury. NMPs have two fates, which can develop into spinal cord and paraxial mesoderm. Recently, research showed that the cells first take the axial orientation, and then accept the neural fate corresponding to the axial level, so that the spinal cord cells will not pass through the intermediate state of the brain [21][. Polevoy and colleagues reevaluated the role of BMP, FGF and Wnt signaling, which are considered to be neural transforming factors, in the development of Xenopus [22]. Their results also support the theory of independent embryonic origin of brain and spinal cord. At present, based on the understanding of the molecular mechanism of NMP formation, a number of studies have successfully differentiated hPSCs into NMPs in vitro, and thus differentiated into more posterior spinal cord neurons [23]. These studies provide a new in vitro model for the assessment of spinal cord developmental toxicity of environmental chemicals.

The neural tube is defined by its anterior posterior axis, but it also has a dorsal-ventral dimension. Dorsal ventral (DV) patterning is essentially a process in which cells located in different positions of DV axis obtain different fate of differentiation. Although it is known that Wnt, BMP from dorsally located epidermis and Shh emanated from the ventrally located notochord are the main morphogen affecting the DV patterning. However, DV regionalization of early embryonic brain is not obvious, and its potential model remains elusive. Recently, researchers propose a model that human forebrain DV patterning also follows an activation/transformation paradigm [24]. They propose that human neuroectoderm (NE) will activate a forebrain dorsal fate automatically, while Shh represses dorsal genes of human NE and subsequently transforms the primitively activated dorsal fate ventrally in a repression release manner. For the spinal cord, the anatomically simplest and most conserved region of the CNS, progress has been made in understanding its DV regionalization, especially, ventral patterning of the spinal cord. Shh induces midline neural plate cells to differentiate into floor plate, and induces floor plate cells to synthesize Shh, which forms a concentration gradient of diffusion to dorsal [25]. Shh downstream transcription factors can be divided into two categories. Among them, class II transcription factors, including Nkx6.1, Olig2 and Nkx2.2, can be activated by Shh signal. Class I transcription factors that can be inhibited by Shh signal include Pax6, IRX3, dbx1 and dbx2. The inhibition between class I and class II proteins resulted in the formation of five types of neural precursor cells with clear boundaries from dorsal to ventral: P0, P1, P2, PMN and P3. With the development, V0–3 interneurons and motor neurons were formed in the ventral side of spinal cord.

## Current methods for derivation of neural organoids

### Current brain organoid methodologies

**Fig. 1:**
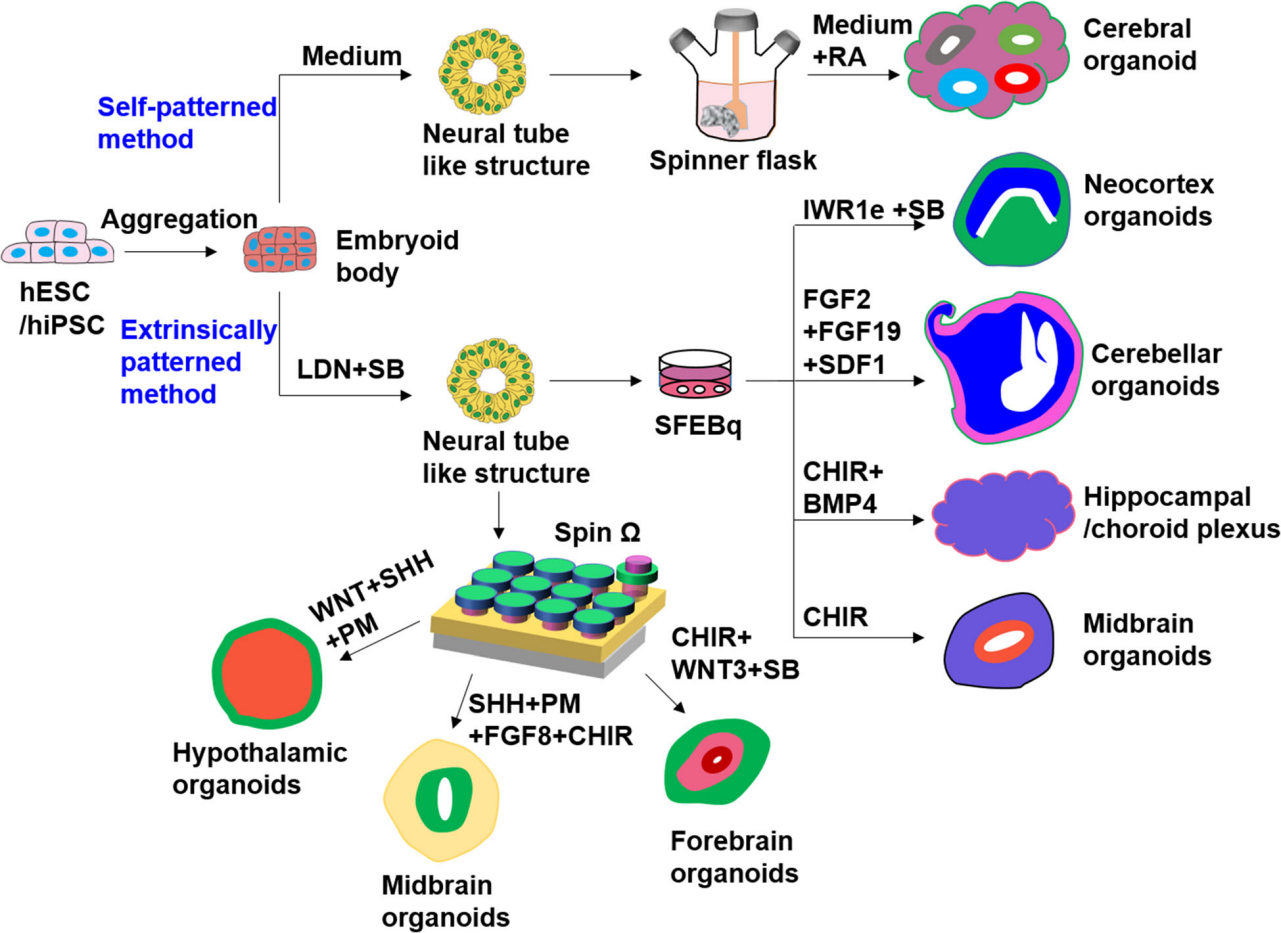
The diagram summarizes the usual process and methods to generate brain organoids. hPSCs firstly divide and aggregate into EBs, which are placed in neural induction media with or without addition of pattering factors to induce neural tube like structures. After expansion, these tissues will be transferred into different devices such as spinning bioreactor, SFEBq culture plate or SpinΩ for specific brain region differentiation and further maturation

Brain organoids can be divided in two categories based on their generating methodologies: self-patterning and extrinsically patterning methods [26]. Self-patterning method refers to organoids cultured without the addition of exogenous pattering factors. By this method, we can obtain the whole-brain organoid, which is a heterogeneous structure with multiple brain regions. Whereas extrinsically patterned methods require addition of external patterning factors to generate the organoid with specific brain region. Figure 1 summarizes the methods of generating different brain regionals identities recapitulated in brain organoids. External patterning factors includes morphogens and neurotrophic factors such as FGF, Shh, Nodal, Wnt and BMP, which can stimulate specific signaling pathways to mimic in vivo development [27].

Lancaster and Knoblich firstly created whole-brain organoid or cerebral organoids containing numerous, but discrete brain regions per organoid using a novel spinning bioreactor approach [28, 29]. This protocol relied solely on the self-organization and intrinsic differentiation of embryoid bodies (EBs) suspended in essential medium. Then, neuroectodermal tissue will form along the outer surface of the EBs and were embedded in Matrigel droplets for neuroepithelial budding [29]. Extracellular matrix (ECM) scaffold improves the apicobasal expansion of the layer of neuroepithelial cells and budding of neuroepithelium. These buds can spontaneously develop into different brain regions ranging from forebrain, midbrain and hindbrain, to retina, choroid plexus and mesoderm without the addition of exogenous patterning factors [30]. The whole brain organoids generated in these studies undergo early stage neural tube formation, neuroepithelium differentiation, and eventually developing into distinct regions, recapitulating some of early features of the human brain development. However, self-patterning method also leads to high variability, heterogeneity, and differentiation of some cells into non-ectodermal cells, posing an obstacle for systematic and quantitative studies for neurotoxicity testing based on whole brain organoids a great challenge.

Currently, most protocols optimize brain organoid methodologies by applying exogenous cues to mimic endogenous patterning. Yoshiki Sasai group first proposed the method to generate brain organoids with specific region from mESCs. They developed a series of 3D differentiation protocols by the serum-free floating culture of EB-like aggregates with quick re-aggregation (SFEBq) technique [31, 32]. In SFEBq protocol, ES aggregates differentiated into neuroectoderm like tissue, and then cortical neurons [31]. Cortical neurons self-organize in a way was similar to early cortical formation. After optimization, self-organized human neocortex was generated by using IWR1e (Wnt inhibitor) and SB (TGFβ inhibitor) in 3D culture [33]. Later, Anca et al. made progress on human cortical spheroids from iPS without any ECM and using the least patterning factors [34]. These spheroids contained deep cortical neurons and superficial cortical neurons. Transcriptional analysis showed that after 2.5 months of culture, these cortical spheroids were similar to the human fetal brain developed in vivo. Muguruma et al. also reported a polarized cerebellar structure self-organizes in 3D SFEBq culture [35]. They found that addition of FGF19 promoted spontaneous generation of dorsoventrally polarized neural-tube-like structures at the level of the cerebellum. Sequential addition of FGF19 and SDF1 induced the generation of continuous cerebellar plate neuroepithelium that differentiated into a multilayered structure as seen in the cerebellar ontogenesis. They further modified the differentiation protocols for the generation of hippocampal-choroid plexus organoids patterned with BMP and Wnt [36]. SFEBq culture method was also used to generate human midbrain organoids by using the dual SMAD inhibition and Wnt activation [37]. Midbrain organoids could secrete dopamine and show functional synapses. Xuyu Qian and colleagues developed a low-cost, easy-to-operate, miniaturized spinning bioreactor (SpinΩ) for brain organoids culture through 3D printing technology [38]. By this device, various region-specific organoids have been successfully developed, for example the forebrain, midbrain, and hypothalamus via the addition of specific patterning factors, further advancing in vitro method for neurotoxicity testing assessment of chemicals. Recently, Teresa et al. reported a new approach for reproducible and scalable generation of cerebellar organoids from hiPSCs by using single-use vertical wheel bioreactors [39]. They equipped bioreactors with a large vertical impeller, which in combination with a U-shaped bottom. Shear distribution inside the vessel is more homogeneous, allowing gentle, uniform mixing and particle suspension with reduced agitation speeds. By this method, they obtained shape and size-controlled cell aggregates, which could generate more homogeneous mature cerebellar organoids. Moreover, they could obtain a larger number of iPSC-derived organoids in a less laborious manner. This scalable system provides a valuable tool for the application of cerebellar organoids in high-throughput screening of neurotoxins. From these studies based on bioreactors, we can see that combination with engineering technology is important for scalable generation of high-quality neural organoids that is critical for toxicity testing applications.

### Current spinal cord organoid methodologies

**Fig. 2:**
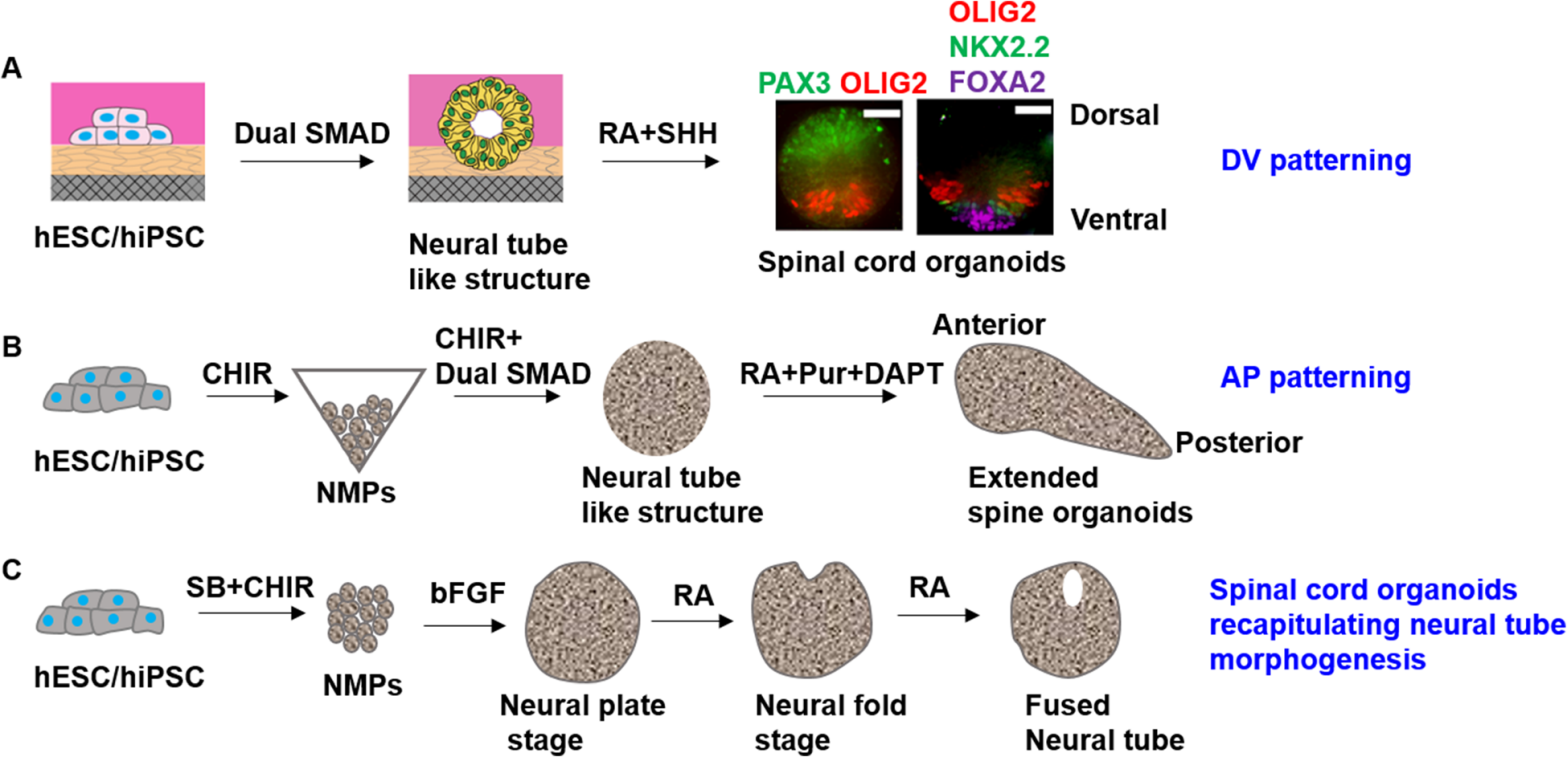
Advances in DV patterning, AP patterning and morphogenesis of neural tube based on formation of spinal cord organoids from hPSCs. (A) In response to Shh and RA signaling, proper dorsal ventral patterned anterior spinal cord organoids were generated from hPSCs in Gel-3D culture system. (B) Through NMPs stage, hPSCs were differentiated into posterior neural tube like structure, which extended into AP patterned spinal organoid by CHIR. (C) Based on advanced 3D culture system, the morphogenetic processes of neural tube formation including polarized neuroepithelial cell induction, sheet-like neural plate formation, and folding-based tube morphogenesis was simulated. Under the action of signal molecules added, fused neural tube like structure was differentiated into spinal cord organoid

In the past years, based on the deepening understanding of the mechanism of spinal cord development, researchers have successfully differentiated hPSCs into various types of spinal cord cells, such as spinal motor neurons and differentiation of V2a interneurons [40, 41]. When researchers tried to culture hPSCs in 3D system, spinal cord organoids were generated by using the same morphogenetic factors as in the induction of spinal cord cells. A summary of advances in spinal cord organoids derived from the differentiation of human PSCs is presented in Fig. 2.

Tanaka group firstly constructed a 3D neural tube model, called neuroepithelial cysts, which could be patterned to spinal cord organoids in vitro using single mouse embryonic stem cells [42]. This study provided a technical basis and research direction for in vitro reconstruction of spinal cord organoids. After that, spinal cord organoids from hPSCs have sprung up. Ribes and colleagues synthesized dorsal spinal cord organoids from mice and humans PSCs [43]. In both species, BMP4 concentration, exposure time or time point determined the type of dorsal cells produced, but the time window of human response to BMP was prolonged. Overall, this study supported the guiding role of BMPs in spinal cord dorsal patterning. Human spinal cord organoids synthesized by Hor et al. are similar to ventral patterned spinal cord in vivo in many aspects [44]. Firstly, the spinal cord organoids can generate motor neurons, excitatory V2a interneurons, inhibitory Renshaw interneurons and astrocytes. Second, these organoids can be patterned along anterior posterior axis. In the absence of exogenous GDF11, they observed HOXB4+ humerus and Hoxc8+ thoracic spinal cord cells. When cultured with mouse myotubes, these ventral spinal cord organoids can form neuromuscular connections and myotube contraction could be observed, proving the function of these organoids. Most importantly, they demonstrated that the ventral spinal cord organoids could mimic the pathology of motor neuron disease Spinal Muscular Atrophy. Ogura et al. synthesized separate dorsal, intermediate and ventral spinal cord organoids to simulate human spinal cord tissue development by regulating the concentration of BMP4 and Shh to control the differentiation direction [45]. However, all of these organoids failed to form the dorsal ventral patterning, which is such an important process for the development of spinal cord.

Recently, Zheng et al. developed a human neural tube model, which could develop into dorsal ventral patterned spinal cord organoid from hPSCs based on a Gel-3D culture system (Fig. 2A) [46]. This culture system integrated some key neurogenic niche elements in vivo, including a 3D basement membrane ECM and a soft gel bed, to reconstruct the mechanical microenvironment of neuroepithelium during neurogenesis. After adding dual SMAD inhibitors, hPSCs self-organized to 3D neural tube like tissues. In response to Shh and RA signaling, neural tube like tissues formed dorsal ventral pattern spinal cord organoids, which were characterized by the adjacent distribution of ventral FP, p3 and pMN domains, while Pax3 positive region was gradually limited to the opposite dorsal pole. This study further proved that the progenitor domains of in patterned spinal cord organoids has the ability to differentiate into neuron domains.

Anterior-posterior body axis patterning of neural tube is another process that is critical for spinal cord development. Researchers have recognized that the posterior neural tube is partially generated from a unique pool of axial stem cells called NMPs. However, the model of neural tube axial elongation is still lacking, which has prevented understanding specifically how NMPs regulate and coordinate the emergence of the posterior spinal cord in humans. Currently, Libby et al. reported AP patterned spinal organoid model that displayed regionalized rostral-caudal HOX gene expression, with spatially distinct hindbrain (HOXB1+) expression from brachial (HOXC6+) and thoracic (HOXB9+) regions (Fig. 2B) [47]. The results demonstrated that Wnt agonism stimulated singular axial extensions in a dose-dependent manner. This spinal organoid model shows great potential for understanding the molecular mechanisms and cellular behaviors that regulate spinal cord development and of course for neurotoxicity testing of chemicals that may result in severe congenital abnormalities, such as spina bifida.

As described above, spinal cord is formed via sequential morphogenetic processes including polarized neuroepithelial cell induction, sheet-like neural plate formation, and folding-based tube morphogenesis (Fig. 2C) [48]. However, the spinal cord organoids above do not mimic morphological features of neural tube formation. Ju-Hyun et al. recently reported an advanced 3D culture system for the production of human spinal cord-like organoids (hSCOs), which exhibited many aspects of spinal cord development, especially neurulation-like tube-forming morphogenesis, as well as differentiation of the major spinal cord neurons and glial cells, and mature synaptic functional activities [49]. The authors further demonstrated that hSCOs platform allowed quantitative and systematic high-throughput examination of the potential risk of neural tube defects induced by antiepileptic drugs. This study advances the application of spinal cord organoids in robust quantification-based neurotoxicity testing.

As mentioned above, great progress has been made in the construction of neural organoids. However, due to the limitation of culture technology and incomplete understanding of neural differentiation mechanism in vivo, these organoids still have their own shortcomings. In Table 1, we summarized the advantages, limitation of neural organoids mentioned above, and their current application in neurotoxicity testing of environmental chemicals, drug for disease treatment and virus. These studies on applications of neural organoids in neurotoxicity testing can undoubtedly provide reference for the neurotoxicity screening of environmental compounds. Next, we will introduce the application progress of neural organoids in the toxicity testing of environmental compounds in detail.

**Table 1:** The advantages, limitations and applications in neurotoxicity testing of various neural organoids

Organoid types	Methods	Advantages	Limitations	Applications in neurotoxicity testing
Cerebral organoid	No patterning factors; Shaking plates	Complete neurogenesis procedure; Various brain in single model; Genetic manipulation	Limited size; Lack of cortical plate; High variability; Lack vasculature	Alcohol [50]; Tranylcypromine [51]; Vincristine [52]; SARS-CoV-2 virus [53];
Neocortex organoid	Extrinsic-patterning factors; Floating culture	Recapitulating second-trimester neocorticogenesis; Emergence of complex separation of cortical zones;	Not recapitulating the morphological separation of all neuronal layers (II/III–VI); High variability; Lack vasculature	Cocaine [54];
Cerebellar organoid	Extrinsic-patterning factors; Floating culturee/ Vertical wheel bioreactors	Recapitulating cerebellar primordium development in first trimester; High reproducibility; Compliant with high-throughput screening (HTS);	Lack vasculature	_
Hippocampal /choroid plexus	Extrinsic-patterning factors; Floating culture	Containing functional hippocampal granule- and pyramidal-like neurons; High efficiency;	Not recapitulation of neural circuitry; Lack vasculature	SARS-CoV-2 [55];
Forebrain organoid	Extrinsic-patterning factors; SpinΩ	Better recapitulating developing human cortex; High reproducibility; Compliant with high-throughput screening (HTS);	Lack vasculature	Zika,BPA [38]; Alcohol [56]; Cd [57];
Midbrain organoid	Extrinsic-patterning factors; SpinΩ/ Floating culture	Better recapitulating developing human cortex; High reproducibility; Compliant with high-throughput screening (HTS);	Lack vasculature	6-hydroxydopamine [58]; A library of 84 compounds [59];
Hypothalamus organoid	Extrinsic-patterning factors; SpinΩ	Better recapitulating developing human cortex; High reproducibility; Compliant with high-throughput screening (HTS);	Lack vasculature	_
Spinal cord	Extrinsic-patterning factors; Floating culture	Partly recapitulating DV and AP patterning of neural tube	Not full patterning; High variability; High tissue heterogeneity; Lack vasculature	6 antiepileptic drugs [49];

## Application of neural organoids in neurotoxicity testing

Given the similarity between neural organoid models and early development of CNS, they may be the most suitable models for studying the chemicals that will cause developmental disorders in fetus. With the development of hPSC-based technologies to generate neural organoid, some recent studies have shown potential for neurotoxicity testing assessment of chemicals based on neural organoids. Table 2 highlights the neural organoid role for neurotoxicity testing of environmental chemicals based on neural organoids.

**Table 2:** Summary of neurotoxicity testing research about environmental chemicals based on neural organoids

Environmental factors	CNS region	Organoid type	Main findings	Reference
60 compounds	Forebrain	Neural constructs	Correctly classified 9 of 10 chemicals in the blinded trial.	[60]
BPA	Forebrain	Forebrain organoids	Dose-dependent decrease in the relative VZ thickness	[38]
TBBPA	Midbrain	Morebrain organoids	TBBPA is a selective toxicant for dopaminergic neurons	[59]
Alcohol	Forebrain, Hindbrain	Brain organoids on an array chip	Attenuated neurite outgrowth and skewed neural maturation; Altered GSX2 gene, RSPO2 gene, and the Hippo signaling pathway	[56]
Cd	Forebrain, Hindbrain	Brain organoids on an array chip	Induced cell apoptosis, skewed neural differentiation, and varied brain regionalization	[57]
Alcohol	Whole brain	Cerebral organoid	Induced cell apoptosis in a dose- and brain cell type-dependent manner; Mitochondrial dysfunction; Ultrastructure changes of cells such as degenerated synapse.	[61]
6 antiepileptic drugs	Spinal cord	Spinal cord organoids	VPA and CBZ resulted a failure of neural tube closure in a dose-dependent manner	[49]

Schwartz et al. developed a 3D brain organoid model that provide a faster, cheaper and more biologically relevant way to screen drugs and chemicals that could harm the developing brain [60]. The brain organoids they developed are the first 3D model including blood vessels and microglia from hPSCs. They cultured neural progenitor cells, vascular cells and microglia derived from stem cells on engineered hydrogels. These precursor cells spontaneously assemble into 3D brain organoid, which possess many characteristics of the developing human brain. Then the researchers treated the neural tissues with 60 different compounds including safe compounds and known toxins. Machine learning was used to build a predictive model from RNA sequencing data. The model was able to accurately classified 9 of 10 chemicals in the blinded trial. This study points out a new direction for high throughput screening (HTS) of neurotoxicity of environmental chemicals.

Xuyu Qian et al.demonstrated the forebrain organoids platform is amenable to chemical compound testing. They tested the effect of Bisphenol A (BPA), which is commonly found in household plastic products and is known to affect rodent neural development [62]. The results showed that BPA led to a dose-dependent decrease in the relative VZ thickness of forebrain organoids after treating with BPA from days 14 to 28. At higher BPA concentrations, decreased density of EdU+ or PH3+ neural progenitor cells (NPCs) was observed, showing that reduced NPC proliferation contributes to decreased relative VZ thickness. This study on the mechanism of BPA demonstrated that the forebrain organoid system allowed quantitative investigation of consequences of BPA exposure. Moreover, two rounds of patterning factors were used to induce forebrain differentiation and significantly reduced both tissue and temporal development heterogeneity. Therefore, their forebrain organoids show high repeatability, which is very important to realize its prospect as a standardized model for neurotoxicity testing.

Pregnant women drinking alcohol during pregnancy can seriously affect the development of fetus brain, leading to a series of symptoms, such as cognitive impairment and psychosis, known as fetal alcohol spectrum disorder, which pathophysiology and mechanism are still unclear. Based on human brain organoids-on-a-chip, Yujuan et al. explored the effects and mechanisms of alcohol exposure on fetal brain development [56]. The brain organoids were generated without the addition of the extra factors. The results showed that the system they developed can high throughput realize controllable formation of embryoid body and in situ development of brain organoids, greatly simplifying the traditional method of brain organoids formation. They observed that alcohol exposure could cause attenuated neurite outgrowth, imbalance of excitatory neurons and inhibitory neurons, and skewed neural maturation. Meanwhile, transcriptome analysis showed abnormal expression of a series of genes related to Hippo signaling pathway, whose abnormal may involve fetal brain malformation, suggesting that alcohol exposure is closely related to fetal brain development disorder. This study provides a simple and robust platform for toxicity evaluation of environmental chemicals in vitro. For example, they also used this brain organoids-on-a-chip system to investigate the neurotoxicity of industrial metal Cd, indicating the presence of impaired neurogenesis in the human fetal brain under Cd exposure [57]. Recently, Arzua et al. attempted to quantify the downstream toxic effects of alcohol exposure on neural pathology phenotypes and signaling pathways within cerebral organoids [61]. The results showed that neurons were more susceptible to alcohol-induced apoptosis than astrocytes. Alcohol exposure organoids showed ultrastructural changes, mitochondrial dysfunction and metabolic stress. Bioinformatic analysis showed that alcohol exposure could lead to changes in expression of genes related to neurodevelopment, and/or implicated in nervous system physiology and neurodegeneration. This human brain model allowed in-depth analyses of neurotoxicity at tissue, cellular, subcellular, metabolism, and gene levels.

Spina bifida, one type of NTDs, caused by the failure of spinal neural tube closure, which can be a result of environmental factors exposure such as antiepileptic drugs. Ju-Hyun et al. analyzed the effects of 6 antiepileptic drugs with three different concentrations on the NTDs based on spinal cord organoids, which could recapitulate in vitro neurulation-like tube-forming morphogenesis [49]. The results showed that valproic acid and carbamazepine treated groups exhibited a failure of neural tube closure in a dose-dependent manner, while others exhibited normal morphogenesis. The study revealed that the spinal cord organoid platform allowed quantitative and systematic high-throughput examination of the potential risk of neural tube defects.

Although neural organoids promise to develop next-generation high-throughput screens that can provide more physiologically relevant predictions of neurotoxicity. However, due to the heterogeneity of organoids, it is difficult to distinguish and quantify the data obtained from organoids we wanted. Henrik et al. developed an automated approach for generation, maintenance, and optical analysis of human midbrain organoids in standard 96-well-plates by using robotic technology [63]. The workflow of the robots include all liquid handling steps (seeding, maintenance, and fixation of organoids) and testing organoids at different stages of development. The automation of the entire workflow enables high-throughput-compatible production of homogenous and reproducible midbrain organoids. They demonstrated that midbrain organoids derived from neural precursor cells displayed a distinctly lower variance compared with iPSC-based organoids in a broad set of parameters, including size, cellular subpopulations, and survival in toxicity studies. Using the automated midbrain organoids generation system, Henrik et al. could simultaneously evaluate the effect of compounds on viability and cellular composition based on midbrain organoids [59]. They screened the general neurotoxic and dopaminergic neuron-specific toxic effects of a library of 84 compounds as a proof-of-principle for high throughput campaigns. In this study, flame retardant 3,3′,5,5′-tetrabromobisphenol A (TBBPA) was firstly identified as a selective toxicant for dopaminergic neurons in human midbrain organoids. Compared with other existing toxicity testing model systems, their organoid workflow provides several obvious advantages: offering higher sensitivity and more physiologically relevant results than standard 2D monocultures or simpler aggregates, quantification of overall toxicity and specific cell types and easily adaptable approach for other cell types. The improvement in equipment and imaging can drastically increase the throughput of the entire workflow.

The more and more publications of neural organoid-based toxicity testing studies of environmental chemicals demonstrating neural organoids have more extensive prospect. However, these studies utilized different protocols to generate organoid even from the same region. Therefore, defining and standardization the conditions required for the derivation of neural organoid with specific CNS region is critical for their application in toxicity testing. In parallel, combination with engineering technologies such as automation technology, for robust and reproducible generating homogeneous neural organoids at large scale is also important for application of neural organoids in toxicology testing.

## Limitation and future perspectives

There are profound species differences in CNS between human and animals, which proves that human neural organoids are superior to animal models. However, like all in vitro models, neural organoids are not identical copies of their in vivo counterparts. Next, we discuss the aspects of neural organoids that are still challenging, which also limit their application in neurotoxicity testing of environmental chemicals, and discuss how current and future advances may help to solve the questions.

The lack of blood-brain barrier in neural organoids may have an important effect on understanding the real toxicity and mechanism of chemicals. Many chemicals cross the blood-brain barrier, but some cannot. Although co-culture with vascular endothelial cells may form vascular networks in brain organoid and 3D biomaterial environments, the problems are that vascular-like networks may not penetrate into human brain organoids, the formed networks may not functional, and the existence of blood vessels may interfere with the self-organization of human brain organoids [64, 65]. Future work to transplant organoids encapsulated in bioactive biomaterials into animals, allowing the host vasculature to grow into the organoid graft, may provide a promising approach to achieve vascularization of human neural organs [66].

In addition, the lack of blood vessels limits the transport of oxygen and nutrients in neural organoids, resulting in restricted growth and maturation of organoids. Recently, Stefano Giandomenico and colleagues developed a method for growing a slice of cerebral organoids on a porous supporting membrane such that the tissue is at the air-liquid interface [67]. This allows cerebral organoids exposed to both the nutrient-rich culture medium below and the oxygen in the air above. This method can help to maintain the mini-brain model cultured for longer time, so that it could mature further. This study has pointed out a new direction for the maturation of neural organoids, which is critical for modeling adult CNS.

Variability and reproducibility of current neural organoids remains the fundamental limiting factor for organoids’ application in neurotoxicity testing. As mentioned above, region-specific brain organoids generated by patterned protocols showed greater organoid-to-organoid reproducibility both differentiation and cellular composition. However, the method rely on autonomous cell fate patterning in a relatively homogenous culture environment, and thus the efficiency of generating patterned organoids remains relatively low [42, 68]. This limits the application of neural organoids for high-throughput neurotoxicity testing, which dependent upon robust and reproducible standardized protocols for generating homogeneous populations of neural organoids at large scale. According to the advances in bioengineering techniques, this limitation expected to be overcome by precise controls of key experimental parameters in a synthetic microfluidic system, which could create an in vivo-like spatiotemporal morphogen gradient [69].

High throughput toxicity screening is a process running millions of environmental chemical tests in a short time to determine their toxic effects. The advantage of high throughput screening is simplicity, rapidness, low cost, non-labor-intensive, high efficiency, as well as leading to a higher information harvest. However, the establishment of high throughput screening approach is accompanied by its own set of challenges. In addition to the above limitations such as vascular deficiency and heterogeneity, the current neural organoid technology still faced with the shortcomings of manual and less intelligent, which makes the efficient, unbiased, high throughput evaluation of neurotoxicants effects by organoid systems challenging. Automation and standardization of cell seeding, maintenance and fixation of organoids can reduce manual intervention and maintain culture process consistency, enabling homogeneous and reproducible neural organoids generation for screening applications. Moreover, Technological advances in high content imaging will be undoubtedly favorable to the application of neural organoids in neurotoxicity screening. Therefore, the establishment of automation neural organoids culture platforms by advanced engineering and robotic technologies will promote organoid’s utility in high throughput screening of neurotoxicants.

## Conclusions

In summary, various studies have shown that neural organoids can recapitulate some key features of the human brain and spinal cord, including cellular distribution and organization, physiological structure, and neuronal networks. Therefore, neural organoids have become a unique model to evaluate the effects of acute and chronic toxin exposure. However, we should recognize the differences between neural organoids and tissues in vivo, such as the lack of blood-brain barrier and spatiotemporal morphogen gradient. The advances for high-fidelity organoids-generating techniques are driving application of neural organoids in neurotoxicity testing.

## Data Availability

Data and materials related to this work are available upon request.

## Abbreviations

2D: Two-dimensional
3D: Three-dimensional
ADHD: Attention Deficit Hyperactivity Disorder
AP: Anterior Posterior
BPA: Bisphenol A
CNS: Central Nervous System
DV: Dorsal Ventral
EB: Embryoid Bodies
ECM: Extracellular Matrix
hESCs: Human Embryonic Stem Cells
hiPSCs: Human Induced Pluripotent Stem Cells
hPSCs: Human Pluripotent Stem Cells
hSCOs: Human Spinal Cord-Like Organoids
HTS: High-Throughput Screening
NE: Neuroectoderm
NTDs: Neural Tube Defects
NMPs: Neural Mesodermal Progenitors
RA: Retinoic Acid
SFEBq: Serum-Free Floating Culture Of EB-Like Aggregates With Quick Re-Aggregation
TBBPA: 3,3′,5,5′-tetrabromobisphenol A
